# Long-term effects of intensive multifactorial treatment on aortic stiffness and central hemodynamics after 13 years with screen-detected type 2 diabetes: the ADDITION-Denmark trial

**DOI:** 10.1186/s13098-022-00890-1

**Published:** 2022-08-17

**Authors:** Lasse Bjerg, Esben Laugesen, Signe Toft Andersen, Jonas Frey Rosborg, Morten Charles, Dorte Vistisen, Daniel R. Witte

**Affiliations:** 1 Steno Diabetes Center Aarhus, Aarhus University Hospital, Hedeager 3, 8200 Aarhus N Aarhus, Denmark; 2 Department of Public Health, Aarhus University, Aarhus, Denmark; 3 Viborg Regional Hospital, Viborg, Denmark; 4 Department of Endocrinology and Internal Medicine, Aarhus University Hospital, Aarhus, Denmark; 5 Danish Pain Research Center, Department of Clinical Medicine, Aarhus University, Aarhus, Denmark; 6 Department of Internal Medicine, Gødstrup Regional Hospital, Herning, Denmark; 7 Research Unit of General Practice, Aarhus University, Aarhus, Denmark; 8 Clinical Epidemiology, Steno Diabetes Center Copenhagen, Herlev, Denmark; 9 Department of Public Health, University of Copenhagen, Copenhagen, Denmark

**Keywords:** Multifactorial treatment, Pulse wave velocity, Type 2 diabetes

## Abstract

**Background:**

Peripheral and central hemodynamic indices are modifiable by lifestyle and medical intervention. We aimed to determine the long-term effect of intensive multifactorial treatment on peripheral and central hemodynamic indices among people with screen-detected diabetes.

**Methods:**

Between 2001 and 2006, people with screen-detected type 2 diabetes were included in the Anglo-Danish-Dutch study of Intensive Treatment of Diabetes in Primary Care (ADDITION) trial (NCT00237549, ClinicalTrials.gov). In the Danish arm, participants were invited to a clinical examination in 2015–2016, 13 years after inclusion and 8 years after trial-end. Out of 586 eligible participants who attended the clinical examination, 411 had a valid examination of central and peripheral hemodynamic indices (242 received intensive treatment and 169 received routine care). Carotid-femoral pulse wave velocity (cfPWV), central blood pressure and augmentation index were assessed by applanation tonometry. We used mixed-effect models to examine the intervention effect adjusting for cluster randomization and heart rate.

**Results:**

Randomization to intensive treatment during the trial-period was associated with a 0.58 m/s lower cfPWV (95% CI − 1.09 to − 0.06) at follow-up. Adjustment for blood pressure attenuated the association. Differences between intervention groups for central augmentation index were − 1.25% (95% CI: − 3.28 to 0.78), central pulse pressure − 1.74 mmHg (95% CI − 4.79 to 1.31), central systolic blood pressure − 3.06 mmHg (− 7.08 to 0.96), and central diastolic blood pressure − 1.70 mmHg (− 3.74 to 0.34).

**Conclusions:**

Intensive multifactorial treatment of screen-detected type 2 diabetes has a sustained positive effect on aortic stiffness measured by cfPWV. Although all estimates pointed in favor of intensive treatment, we observed no clear beneficial effect on other hemodynamic indices.

**Supplementary Information:**

The online version contains supplementary material available at 10.1186/s13098-022-00890-1.

## Background

Cardiovascular disease (CVD) is a frequent complication of type 2 diabetes [1] leading to higher cardiovascular risk in people with type 2 diabetes compared with the background population [2, 3]. Clinical trials have shown that the development of CVD and CVD mortality can be reduced by multifactorial intervention, both during the intervention window and up to several years after its cessation [4, 5]. This legacy effect of multifactorial intervention on CVD was corroborated by results from The Anglo-Danish-Dutch Study of Intensive Treatment in People with Screen Detected Diabetes in Primary Care (ADDITION) [6]. Ten years after the intervention, the ADDITION-trial reported a 13% (HR = 0.87, 95%CI 0.73–1.04) statistically non-significant cardiovascular risk reduction in people with screen-detected type 2 diabetes attending general practices randomized to provide intensive care compared with people attending general practices randomized to provide routine care [7]. This result should be seen in the light of the small differences between the two randomization groups in levels of HbA1c, lipids and blood pressure at trial end and almost identical levels of HbA1c, lipids and blood pressure in the two groups at 10 years follow-up [7, 8], while both groups saw a marked improvement in risk factor levels from the trial baseline.

Aortic stiffness, measured as carotid-femoral pulse wave velocity (cfPWV), is an independent predictor of CVD and mortality [9, 10]. cfPWV is regarded the gold-standard method for non-invasive assessment of aortic stiffness [11] that can be measured with relatively simple equipment. cfPWV is increasingly used as an intermediate cardiovascular outcome in research in both individuals with and without diabetes [10, 12, 13]. Central blood pressure and central pulse pressure reflect the pressure in the ascending aorta and are highly relevant for the pathogenesis of CVD [14] while the augmentation index represents an indirect measure of wave reflections, one of the determinants of aortic stiffness [15]. Both central blood pressure, pulse pressure, and augmentation index are associated with increased risk of CVD [12, 14, 15].

Intensive risk factor management with antihypertensive and lipid-lowering treatment has been shown to reduce aortic stiffness [16–19]. Also, a contemporaneous effect of intensive multifactorial treatment on arterial stiffness in people with screen-detected type 2 diabetes, assessed by cfPWV, was observed in the ADDITION-Denmark at the end of the trial five years after inclusion [20]. Despite the positive impact of well-conducted risk factor management on both CVD and cfPWV in people with diabetes, it is unknown whether a sustained effect of intensive multifactorial treatment on cfPWV exists in people with screen-detected type 2 diabetes.

We hypothesized that intensive multifactorial treatment early in the course of diabetes, including target driven glycemic control, lipid-lowering medication and antihypertensive medication, may have long-term effects on both central and peripheral hemodynamics and reduce the process of arterial stiffening in people with screen-detected type 2 diabetes.

## Methods

### Study design and population

**Fig. 1:**
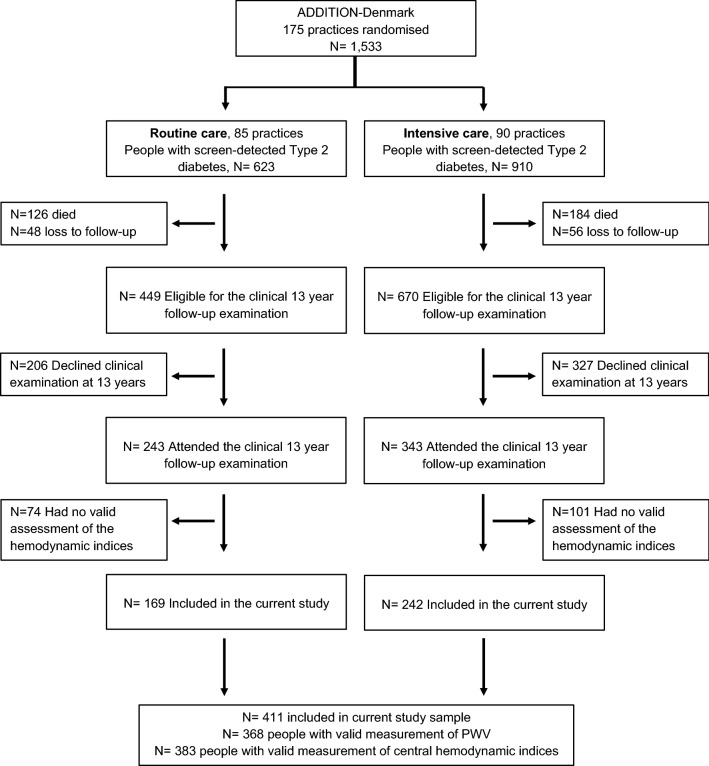
Flow of the included study sample

The current study is a post-hoc analysis of the central and peripheral hemodynamic outcomes from the Danish arm of the ADDITION-trial collected 13 years following randomization. The rationale, methods and results from the cluster randomized-controlled ADDITION-trial have been reported in detail previously [6]. Briefly, following a stepwise screening programme, 190 participating Danish general practices were randomly assigned to provide either routine- or intensive care of diabetes. The intervention was delivered through education of the staff in the general practice and included specific treatment recommendations [6]. In total, the Danish arm of the ADDITION trial included 1,533 participants (aged 40–69 years) with screen-detected type 2 diabetes, included between 2001 and 2006. In 2015–2016, 8 years after trial-end and 13 years after the diagnosis of type 2 diabetes by screening, a clinical follow-up examination was performed at five Danish study Centers, however the central hemodynamic assessment was only performed at four out of five study centers. In total, we obtained hemodynamic measures in 411 out of 586 attending people, who constitute the study sample in the current analyses (Fig. 1). The reasons for the missing data was heterogeneous and were as follows: logistic reasons or insufficient staff (n = 106), technical failure of the Sphygmocor device (n = 13), irregular pulse (n = 9), the carotid or femoral pulse could not be found (n = 10), or attendance at the study center without central hemodynamic assessment (n = 38).

The study was conducted in accordance with the principles of the 1996 Declaration of Helsinki, approved by the local Ethics committee in the Central Denmark Region (approval numbers 20000183 and 1-10-72–63-15), and approved by the Danish Data Protection Agency (approval no. 2005-57-0002, ID185). All study participants gave written informed consent. The ADDITION trial is registered with NCT00237549, ClinicalTrials.gov.

### Covariates

Using standardized operating procedures, a physical health assessment including anthropometrics and venous blood samples was performed at baseline and follow-up. Biochemistry measures included HbA1c, total cholesterol, triglycerides, low-density lipoprotein cholesterol (LDL cholesterol), high-density lipoprotein cholesterol (HDL cholesterol). Albumin-to-creatinine ratio [u-ACR] was measured in spot urine samples. From self-reported questionnaires, we obtained information on smoking status and alcohol consumption, while participants’ general practitioners provided baseline records of prescribed medication. At follow-up prescribed medication was obtained from the Danish National Health Service Prescription Database [21].

### Outcomes

The assessment of central and peripheral hemodynamics was performed by trained staff according to standardized study protocols and has been described previously [20]. cfPWV, central systolic and diastolic blood pressure, pulse pressure, and augmentation index were assessed by the SphygmoCor device (version 8, Atcor Medical) while peripheral hemodynamics were assessed by systolic and diastolic blood pressure as described below.

### Central hemodynamics

Before assessment of central hemodynamic indices, the brachial blood pressure was measured after 5 min of rest with the patient in supine position (Omron M6-AC, Omron Healthcare, Milton Keynes, UK). The mean blood pressure was calculated as diastolic blood pressure + 0.4 × pulse pressure [22].

We assessed the velocity of the pulse waves between the right carotid and right femoral arteries. The travel distance was determined by subtracting the distance from the suprasternal notch to the carotid artery (measured with a tape measure) from the distance from the suprasternal notch to the femoral artery (measured with an anthropometer). Recordings of pulse waves at the carotid and femoral arteries were then assessed by applanation tonometry along with an electrocardiogram. The transit time was determined using the intersecting tangent method [23] and the transit time was defined as the mean of 10 pulse waves. Two consecutive measures of cfPWV were made in each individual. If the measures differed by more than 0.5 m/s, a third measure was performed. For each individual, the average of the two closest measurements of cfPWV was used.

Using the SphygmoCor device and its built-in software, we estimated central systolic and diastolic blood pressure, central pulse pressure, and central augmentation index from peripheral pressure waveforms recorded at the radial artery. Pulse wave analysis data with an operator index < 75 were excluded.

### Peripheral hemodynamics

Brachial systolic and diastolic blood pressure were measured after 10 min of rest in a sitting position with an automated blood pressure recorder (Omron M6-AC, Omron Healthcare, Milton Keynes, UK). The blood pressure was measured three times and the average was used in the analysis. We estimated brachial pulse pressure as the difference between brachial systolic blood pressure and diastolic blood pressure.

### Statistical analysis

We tabulated characteristics of included individuals at baseline, trial-end and at follow-up by randomization group. Data are presented as medians (p25–p75) and proportions (%).

We used linear mixed-effect models (as we accounted for cluster-randomization) to estimate the effect (95% confidence intervals (CI)) of randomization to multifactorial treatment on hemodynamic indices in an intention-to-treat approach. The models were adjusted for clustering at practice level and heart rate at the time of measurement (model 1) and furthermore adjusted for age and sex (model 2). The analysis of cfPWV was further adjusted for mean blood pressure at the time of measurement. We assessed any effect modification by sex by including an interaction term between sex and randomization group. To assess the internal validity of our study sample, we tabulated baseline characteristics and 13 years follow-up characteristics between participants who attended the clinical follow-up examination but did not have an assessment of cfPWV or central hemodynamic indices and our study sample. Furthermore, to assess potential selection bias, we examined cfPWV at five year by randomization group in those attending the 13 years follow-up examination.

Calculations and graphs were made in R version 3.3.3 (R Foundation for Statistical Computing, Vienna, Austria, www.R-project.org).

## Results

In 2015–2016, 1,119 eligible participants were invited to the post-trial clinical follow-up examination nearly 13 years after (median = 12.8 years) inclusion in the ADDITION trial. Measurements of hemodynamic indices were successfully obtained in 411 of the 586 attending participants and this group constitutes the study sample in the current study. Of these, 242 participants attended general practices originally randomized to deliver intensive treatment and 169 participants attended practices randomized to deliverer routine care (Fig. 1).

Characteristics of the study sample at baseline were similar between the intensive treatment group and routine-care group (Table 1). Our study sample had a mean age of 58 years at inclusion and included 35% women. The percentage of participants receiving antihypertensive medication and lipid lowering medication at baseline were 34% and 14%, respectively. The percentage of participants receiving antihypertensive medication and lipid lowering medication was much higher at 13 years follow-up in both randomization-groups. The level of HbA_1c_, BMI, and waist circumference were comparable at baseline and at follow-up. After 13 years of follow-up, cholesterol levels, systolic and diastolic blood pressure and weekly alcohol consumption decreased in both randomization-groups as did the percentages of current smokers. Overall, the intensive treatment group and the routine care groups were similar with regard to medication and levels of observed clinical variables at 13 years follow-up (Table 1).

**Table 1:** Characteristics of the study sample at baseline and 13 years follow-up

	At baseline			At 13 years follow-up		
	Routine	Intensive	N	Routine	Intensive	N
n	169	242		169	242	
Female sex	59 (34.9)	84 (34.7)	411	59 (34.9)	84 (34.7)	411
Age at inclusion (years)	58 (6.8)	58 (6.5)	411	58 (6.8)	58 (6.5)	411
Age at follow-up (years)				71 (6.72)	70 (6.63)	411
Follow-up time (years)				12.6 (1.41)	12.5 (1.42)	411
BMI (kg/m^2^)	31 (4.9)	30 (5.3)	399	30 (5.2)	30 (5.8)	410
Waist (cm)	104.7 (12.2)	104 (12.8)	399	105 (13.2)	106 (14.2)	407
Systolic blood pressure (mmHg)	148.3 (19.3)	145.6 (16.8)	399	141.2 (16.3)	137.4 (15.5)	410
Diastolic blood pressure (mmHg)	88.1 (10.6)	87.6 (9.6)	399	83.6 (9.9)	82.0 (9.3)	410
HbA1_c_ (%)	6.7 (1.4)	6.9 (1.6)	395	6.7 (0.9)	6.9 (0.9)	410
HbA1_c_ (mmol/mol)	50 (15)	52 (17)	395	50 (10)	52 (10)	410
Total cholesterol (mmol/L)	5.8 (1.2)	5.7 (1.1)	384	4.4 (1.0)	4.3 (1.0)	410
Triglycerides (mmol/L)	1.97 (1.3)	1.95 (1.3)	377	1.69 (0.7)	1.82 (0.9)	410
HDL cholesterol (mmol/L)	1.39 (0.3)	1.38 (0.4)	374	1.38 (0.4)	1.37 (0.4)	410
LDL cholesterol (mmol/L)	3.45 (1.0)	3.45 (1.0)	362	2.26 (0.8)	2.16 (0.8)	398
Albumine Creatinine ratio (mg/g)	2.95 (10.5)	1.88 (4.2)	372	8.31 (37.1)	5.89 (20.3)	406
Any glucose-lowering drug	s	–	411	106 (72.6)	149 (77.6)	338
Metformin	–	–	411	91 (62.3)	131 (68.2)	338
Insulin	–	–	411	24 (16.4)	36 (18.8)	338
Sulphonylurea	–	–	411	10 (6.8)	16 (8.3)	338
Antihypertensives	62 (36.7)	79 (32.6)	411	120 (82.2)	165 (85.9)	338
ACE/ARB blockers	25 (14.8)	37 (15.3)	411	101 (69.2)	147 (76.6)	338
Betablockers	24 (14.2)	32 (13.2)	411	39 (26.7)	50 (26.0)	338
Calcium antagonists	17 (10.1)	21 (8.7)	411	57 (39.0)	69 (35.9)	338
Diuretics	35 (20.7)	41 (16.9)	411	66 (45.2)	105 (54.7)	338
Statins	21 (12.4)	35 (14.5)	411	113 (77.4)	155 (80.7)	338
Aspirin	15 (8.9)	24 (9.9)	411	66 (45.2)	119 (62.0)	338
Smoking			407			379
Non-smoker	56 (33.7)	101 (41.9)		54 (34.2)	90 (40.7)	
Former smoker	63 (38.0)	81 (33.6)		79 (50.0)	104 (47.1)	
Current smoker	47 (28.3)	59 (24.5)		25 (15.8)	27 (12.2)	
Alcohol use *	47 (29.2)	66 (29.9)	382	7.7 (8.9)	7.7 (9.0)	308

Categorical data are expressed as n (%), and continuous data as means (SD)

^*^ Weekly alcohol consumption exceeding recommended intake (> 7 units in women and > 14 units in men)

At follow-up, mean cfPWV was 10.5 m/s and 9.9 m/s in the routine care group and intensive treatment group, respectively (Table 2). Differences between the intensive treatment group and the routine care group in brachial systolic blood pressure, brachial mean blood pressure, central systolic blood pressure, and central mean blood pressure were not statistically significant (Table 2).

**Table 2:** Hemodynamic characteristics at 13 years follow-up by treatment group

Hemodynamic indices	Routine care	Intensive treatment
N	169	242
Brachial systolic blood pressure (mmHg)	141.2 (16)	137.4 (15)
Brachial diastolic blood pressure (mmHg)	83.6 (10)	82.0 (9)
Brachial pulse pressure (mmHg)	57.6 (13)	55.4 (13)
Mean brachial blood pressure (mmHg)	106.4 (12)	103.7 (11)
Central systolic blood pressure (mmHg)	131.0 (17)	127.5 (16)
Central diastolic blood pressure (mmHg)	83.5 (10)	81.8 (9)
Mean central blood pressure (mmHg)	103.1 (12)	100.7 (11)
Central pulse pressure (mmHg)	47,5 (13)	45.7 (14)
Central augmentation index (%)	28.5 (10.2)	27.7 (9.4)
Aortic pulse wave velocity (m/s)	10.5 (2.6)	9.9 (2.2)

Data are means (SD)

*N* number of observations

We found that the intensive treatment group had 0.58 m/s lower cfPWV (95% CI − 1.09 to − 0.06 m/s) compared with the routine care group after adjustment for cluster randomization and heart rate. With further adjustment for age and sex, the cfPWV difference was 0.48 m/s (95% CI − 0.97 to 0.01 m/s) in favor of intensive treatment. Accounting for the effects of mean blood pressure the cfPWV difference between treatment groups attenuated further to 0.35 m/s lower (95% CI − 0.84 to 0.14 m/s) in the intensive treatment group. With adjustment for heart rate and cluster randomization, central mean arterial pressure was 2.14 mmHg lower (95% CI − 4.87 to 0.59 mmHg) and central augmentation index was 1.25% lower (95% CI − 3.28 to 0.78%) in the intensive treatment compared with the routine care group. In line with this, we found no statistically significant differences between treatment arms in other central or peripheral hemodynamic indices. After adjustment for age and sex, the associations attenuated, but all hemodynamic estimates still pointed in favor of intensive treatment (Table 3). We found no effect modification by sex.

**Table 3:** Effect of intensive treatment compared with routine care on hemodynamic indices

	Model 1	Model 2
Hemodynamic indices	Estimate (95% CI)	Estimate (95% CI)
Peripheral systolic blood pressure (mmHg)	− 3.38 (− 7.14 to 0.38)	− 3.34 (− 7.12 to 0.43)
Peripheral diasolic blood pressure (mmHg)	− 1.23 (− 3.21 to 0.76)	− 1.47 (− 3.4 to 0.45)
Peripheral pulse pressure (mmHg)	− 2.33 (− 5.28 to 0.62)	− 1.97 (− 4.83 to 0.89)
Central systolic blood pressure (mmHg)	− 3.06 (− 7.08 to 0.96)	− 2.85 (− 6.82 to 1.12)
Central diastolic blood pressure (mmHg)	− 1.70 (− 3.74 to 0.34)	− 1.85 (− 3.85 to 0.16)
Central pulse pressure (mmHg)	− 1.74 (− 4.79 to 1.31)	− 1.28 (− 4.11 to 1.55)
Central mean arterial pressure (mmHg)	− 2.14 (− 4.87 to 0.59)	− 2.19 (− 4.94 to 0.56)
Central augmentation index (%)	− 1.25 (− 3.28 to 0.78)	− 1.03 (− 2.85 to 0.79)
Pulse wave velocity (m/s)	− 0.58 (− 1.09 to − 0.06) †	− 0.48 (− 0.97 to 0.01)
Pulse wave velocity (m/s)*	− 0.47 (− 0.99 to 0.06)	− 0.35 (− 0.84 to 0.14)

Model 1: Adjustment for heart rate at the time of assessment and cluster randomization. Model 2: Model 1 plus adjustment for age and sex

^*^Model 1 and model 2 additionally adjusted for mean blood pressure at the time of assessment. Mean blood pressure was calculated as: peripheral diastolic blood pressure + (0.4* peripheral pulse pressure)

^†^p < 0.05

We assessed characteristics of the study sample by trial-end (5 years after inclusion) (Additional file 1: Table S1). Overall, characteristics were broadly similar in the randomization groups. However, glycose-lowering medication overall, ACE inhibitors or ARB, lipid-lowering drugs and aspirin were more often prescribed in the intensive treatment group than in the routine care group. Also, levels of total cholesterol and LDL cholesterol were lower in the intensive treatment group. (Additional file 1: Table S1).

Baseline characteristics and follow-up characteristics of participants attending the 13 year clinical follow-up examination without assessments of cfPWV and central hemodynamic indices (n = 176) were highly similar to the characteristics of the study sample (those with measures of hemodynamic indices). However, participants in the study sample were a few years younger and were more often prescribed antihypertensive medications at inclusion (Additional file 1: Table S2). After adjustment for cluster randomization and heart rate the cfPWV at 5 year was 0.65 m/s (95% CI − 1.04 to − 0.26 m/s) lower in those who attended the 13 years follow-up compared with those who did not attend the 13 years follow-up. We found that the intensive treatment group had 0.56 m/s lower cfPWV (95% CI − 1.23 to 0.12 m/s) compared with the routine care group in those who did not attend the 13 years follow-up and 0.50 m/s lower cfPWV (95% CI − 1.00 to − 0.00 m/s) in those who did attend the 13 years follow-up.

### Discussion

In this analysis of 411 persons with screen-detected type 2 diabetes, we found that participants who attended general practices randomized to provide multifactorial target driven intensive treatment during the 5-year trial period had lower cfPWV 13 years post-randomization compared to individuals who attended general practices randomized to provide routine care during the trial. In addition, our results indicate a favorable statistically non-significant effect of intensive treatment on the other central and peripheral hemodynamic indices included in this study.

During the ADDITON trial, the care for people with diabetes improved due to a change in the national guidelines for diabetes care. The changes included more aggressive treatment with very similar treatment goals to those used for the intensive treatment group of the ADDITON trial. The changes of the clinical guidelines were largely driven by the results of the Danish STENO-2 trial [4]. Results from STENO-2 pointed towards a clear beneficial effect on CVD risk of multifactorial treatment, including formalized goals for blood pressure using ACE/ inhibitors or ARB and formalized goal for LDL-cholesterol by statin treatment in type 2 diabetes patients with persistent microalbuminuria [24].

It is likely and arguable that changes in clinical recommendations during the trial period affected the intensity of cardiovascular risk factor management in both arms of the ADDITION trial, reducing the difference between the two groups and thus attenuate the intervention effect that we observe. In this light, it is interesting that even the relatively small differences in cardiometabolic risk factors in the intensive treatment arm, against a background of over-all tighter management led to lower arterial stiffness. This observation affirms the role that intermediate end-points, and particularly cfPWV can play in the monitoring of the impact of cardiovascular risk management strategies. We have previously published data on peripheral and central hemodynamic indices at trial-end using data from the ADDITION-Denmark trial [20]. We reported that participants attending practices randomized to deliver intensive treatment had 0.51 m/s lower (95% CI − 0.96–0.05 m/s) cfPWV compared with those attending practices that provided routine care [20]. In the present analysis 13 years after inclusion, we found a sustained effect in individuals attending practices randomized to provide intensive treatment i.e. cfPWV was 0.58 m/s lower compared with individuals attending practices that provided routine care. This persistent effect could conceptually be explained by either a sustained difference in cardiovascular risk factors post-intervention, or by an early effect on arterial stiffness during the trial period which persists after the end of the intervention.

Overall, there was a reduction of most risk factors by the end of the ADDITION trial (at five year follow-up) with some differences between randomization groups [8]. At 10 years follow-up in the international ADDITION trial, the reduction of most CVD risk factors (bodyweight, HbA_1c_, cholesterols and blood pressure) was sustained, but the difference between treatment arms was attenuated or lost [7]. In line with these results, we observed that lipid lowering medication, aspirin, and ACE inhibitors or ARB were more frequently used by the intensive treatment group at trial-end in the sub-sample of the Danish arm of ADDITION included in this analysis. However, except for aspirin, these differences diminished at 13 years follow-up. We do not know when the difference in treatment and risk factors leveled out between the intervention groups, but our results do not support the notion that the lower arterial stiffness observed 5 years after the end of the trial period is due to a sustained difference in risk factor levels, which in turn maintain the difference in cfPWV. Rather, our results are consistent with an early effect of intensive risk factor management on arterial stiffness, which is maintained even when the risk factor differences diminish i.e. we may observe a carryover effect founded during the intervention period. Results from STENO-2 and other clinical trials also report a sustained effect of intervention on diabetes related complications even years after the active intervention has stopped, which corroborates this hypothesis [5, 25].

Our study points towards a small positive effect of multifactorial intervention on all hemodynamic indices. The marked attenuation of the difference in cfPWV observed in the study by further adjustment for the mediating effect of mean brachial blood pressure, supports the notion that blood pressure changes partly mediate the lower cfPWV, although, we did not have power to detect a marked difference between randomization groups in mean arterial blood pressure.

The first line drugs for the management of peripheral hypertension both during the trial period and now are ACE inhibitors or ARBs [26]. They affect the elasticity of the arterial wall by modulation of the kidney function and through direct effects on the wall, which in turn may lead to reduced cfPWV [17, 27]. In line with this, other studies have shown that people both with and without diabetes but treated with an ACE inhibitor or ARB have reduced cfPWV [16, 17]. However, the long-term effect by ACE or ARBs on pulse wave velocity is not clear. Similarly, studies have suggested, that statin treatment lowers aortic stiffness [18, 19]. The exact mechanisms is not clear but reduction of the vascular remodeling and vascular tone in combination with reduced oxidative stress have been suggested [28, 29]. New glucose lowering drugs may decrease pulse wave velocity directly [30]. However, in the ADDITION trial, metformin was the first line drug and there seems to be no direct effect by metformin on pulse wave velocity [31]. Our results corroborates that differences in the use of antihypertensive medication and lipid-lowering medication may reduce pulse wave velocity as the difference in aortic stiffness first observed at the end of the trial period, have now been observed to be sustained.

## Strengths and limitations

The main strengths of the present study are its randomized design and long follow-up. We present a post-hoc analysis of measures that were not part of the registered primary or secondary end-points of the ADDITION-trial, but are conceptually closely related as intermediate measures. The study sample in this analysis is broadly representative of those attending the ADDITION-Denmark clinical examination 13 years after randomization but is selected in comparison to the baseline trial population. Previously, we have assessed differences between those attending the clinical follow-up and those who did not [32]. Those attending the clinical examination were younger and had less comorbidity compared to those eligible who did not attend the clinical 13 years follow-up examination [32]. In contrast, self-rated health and HbA1c levels were similar between those attending and those not attending clinical ADDITION follow-up examination. The cfPWV at five year was higher in those not attending the 13 years follow-up. However, the difference between randomization groups in cfPWV at five year follow-up were highly similar in those attending the 13 years follow-up and those who did not. Thus, we expect the selection introduced during follow-up to be non-differential with regard to this study’s outcome. Overall, we observe cfPWV that are relatively low in both treatment arms at 13 years follow-up and within the normal range of PWV compared to a normal population [33]. In summary, those attending the clinical follow-up had less comorbidity and relatively good cfPWV, which might lead to a small underestimation of the difference between treatment arms. We used a mixed-effect model to be able to account for randomization at practice level. The general practices were not blinded to treatment allocation, as the intervention was delivered through education of the staff in the general practice. Any incomplete adherence to treatment algorithms by general practice staff would presumably attenuate between-group differences in risk factors and treatment, thus leading to reduced differences in hemodynamic indices and cfPWV. Although the hemodynamic indices were not measured at the trial baseline, we believe it is reasonable to assume that they were similar in people attending practices randomized to provide intensive treatment and people attending practices randomized to provide routine care as we observed similar levels of the vast majority of other variables at baseline. Therefore, we assume that any observed differences occurred during the follow-up period.

## Conclusions

Intensive multifactorial treatment, including targeted blood pressure, lipids and HbA1c, does not only have a positive effect on aortic stiffness during intervention in screen-detected type 2 diabetes, it also has a sustained effect at least 8 years after intervention. Notably, any effect of intervention on other peripheral or central hemodynamic indices was less clear, although all estimates pointed in favor of intensive treatment. Lower cfPWV is associated with lower CVD risk. A recent meta-analysis has shown, that 1 m/s lower cfPWV is associated with 12% lower CVD risk and 9% lower CVD mortality [34]. The difference we observe of 0.58 m/s would correspond to an extrapolated 7% CVD risk reduction and an extrapolated 5% CVD mortality risk reduction based on the estimates from the meta-analysis [34]. Our results suggests the positive effect of intensive multifactorial cardiometabolic risk factor management on cfPWV may be mediated through blood pressure. The findings of this study suggest that optimization of treatment early after diagnosis of type 2 diabetes may reduce the development of aortic stiffness, a valid intermediate cardiovascular outcome, in people with screen-detected type 2 diabetes and corroborates the critical importance of early detection and treatment of type 2 diabetes.

## Supplementary Information

**Additional file 1: Table S1**. Characteristics of the study sample at the end of the trial (by 5 years follow-up). **Table S2.** Baseline and at follow-up characteristics of persons with and without hemodymanic assessment at 13 years follow-up

## Data Availability

The datasets generated and analyzed during the current study are available from the senior author (D.R.W.) on reasonable request.

## Abbreviations

ACE: Angiotensin converting enzyme
ADDITION: Anglo-Danish-Dutch study of Intensive Treatment of Diabetes in Primary Care
ARB: Angiotensin receptor blocker
BMI: Body mass index
CI: Confidence interval
cfPWV: Carotid-femoral pulse wave velocity
CVD: Cardiovascular disease
HDL cholesterol: High-density lipoprotein cholesterol
HR: Hazard ratio
LDL cholesterol: Low-density lipoprotein cholesterol
u-ACR: Albumin-to-creatinine ratio
