# Supplementary material for: Long-term effects of intensive multifactorial treatment on aortic stiffness and central hemodynamics after 13 years with screen-detected type 2 diabetes: the ADDITION-Denmark trial
Source: Diabetol Metab Syndr. 2022 Aug 17;14:116. doi: 10.1186/s13098-022-00890-1 (PMC9382804; doi:10.1186/s13098-022-00890-1)
Supplement: Supplementary file 1 — Additional file 1: Table S1. Characteristics of the study sample at the end of the trial (by 5 years follow-up). Table S2. Baseline and at follow-up characteristics of persons with and without hemodymanic assessment at 13 years follow-up [file 13098_2022_890_MOESM1_ESM.docx]

**Long-term effects of intensive multifactorial treatment on aortic stiffness and central hemodynamics after 13 years with screen-detected type 2 diabetes: the ADDITION-Denmark trial**

Lasse Bjerg ^1, 2, 3^, Esben Laugesen ^4^, Signe Toft Andersen ^1, 5, 6^, Jonas Frey Rosborg ^1^, Morten Charles ^1, 7^, Dorte Vistisen ^8, 9^, Daniel R. Witte ^1, 2^

^1^Steno Diabetes Center Aarhus, Denmark

^2^Department of Public Health, Aarhus University, Denmark

^3^Department of Paediatrics, Viborg Regional Hospital, Viborg, Denmark

^4^Department of Endocrinology and Internal Medicine, Aarhus University Hospital, Denmark.

^5^Danish Pain Research Center, Department of Clinical Medicine, Aarhus University, Denmark

^6^Department of Internal Medicine, Gødstrup Regional Hospital, Herning, Denmark

^7^Research Unit of General Practice, Aarhus University, Denmark

^8^Clinical Epidemiology, Steno Diabetes Center Copenhagen, Denmark

^9^Department of Public Health, University of Copenhagen, Capital Region, Denmark

**Corresponding author**

Lasse Bjerg

Steno Diabetes Center Aarhus, Aarhus University Hospital, Hedeager 3, 8200 Aarhus N, Denmark

Phone: +45 2443 4683; e-mail: [lassehan@rm.dk](mailto:lassehan@rm.dk)

Supplementary Table S1

Characteristics of the study sample at the end of the trial (by 5 years follow-up)

|  | By the end of the trial period | |
| --- | --- | --- |
|  | Routine care | Intensive treatment |
| n | 169 | 242 |
| Female sex | 110 (65.1) | 158 (65.3) |
| Age at end of the trial (years) | 64 (6.7) | 63 (6.6) |
| BMI (kg/m^2^) | 30.7 (5.2) | 30.2 (5.3) |
| Waist (cm) | 104.8 (12.7) | 103.8 (13.7) |
| Systolic blood pressure (mmHg) | 135.9 (17.3) | 134.2 (16.1) |
| Diastolic blood pressure (mmHg) | 85.0 (10.1) | 844 (9.8) |
| HbA1_c_ (%) | 6.6 (0.9) | 6.5 (0.9) |
| HbA1_c_ (mmol/mol) | 49 (10) | 48 (10) |
| Total cholesterol (mmol/L) | 4.4 (0.8) | 4.2 (1.0) |
| Triglycerides (mmol/L) | 1.7 (0.9) | 1.7 (1.0) |
| HDL cholesterol (mmol/L) | 1.4 (0.4) | 1.4 (0.4) |
| LDL cholesterol (mmol/L) | 2.3 (0.8) | 2.1 (0.8) |
| Albumine Creatinine ratio (mg/g) | 4.4 (13.2) | 3.7 (10.7) |
| Any glucose-lowering drug | 83 (49.4) | 149 (62.6) |
| Metformin | 73 (43.5) | 120 (50.4) |
| Insulin | 9 (5.4) | 21 (8.8) |
| Sulphonylurea | 25 (14.9) | 35 (14.7) |
| Antihypertensives | 127 (75.6) | 195 (81.9) |
| ACE/ARB blockers | 104 (61.9) | 185 (77.7) |
| Betablockers | 33 (19.6) | 41 (17.2) |
| Calcium antagonists | 54 (32.1) | 74 (31.1) |
| Diuretics | 84 (50.0) | 121 (50.8) |
| Statins | 122 (72.6) | 193 (81.1) |
| Aspirin | 83 (49.4) | 193 (81.1) |
| Smoking | |  |
| Non-smoker | 55 (34.0) | 96 (41.2) |
| Former smoker | 73 (45.1) | 93 (39.9) |
| Current smoker | 34 (21.0) | 44 (18.9) |

Categorical data are expressed as n (%), and continuous data as means (SD).

Supplementary table S2

Baseline and at follow-up characteristics of persons with and without hemodymanic assessment at 13 years follow-up

|  | At Baseline | | At 13 years follow-up | |
| --- | --- | --- | --- | --- |
|  | Without hemodynamic assessment | With hemodynamic assessment | Without hemodynamic assessment | With hemodynamic assessment |
| n | 176 | 411 | 176 | 411 |
| Female sex | 69 (39.4) | 143 (34.8) | 69 (39.4) | 143 (34.8) |
| Age at inclusion (years) | 59 (6.0) | 58 (6.6) | 59 (6.0) | 58 (6.6) |
| Age at follow-up (years) |  |  | 71 (6.0) | 70 (6.8) |
| Follow-up time (years) |  |  | 12.2 (1.6) | 12.5 (1.4) |
| BMI (kg/m^2^) | 31.3 (5.6) | 30.5 (5.1) | 30.9 (5.9) | 30.3 (5.5) |
| Waist (cm) | 105.7 (13.3) | 104.3 (12.6) | 106.1 (14.3) | 105.5 (13.8) |
| Systolic blood pressure (mmHg) | 149.2 (20.7) | 146.8 (18.0) | 138.5 (17.9) | 139.0 (15.9) |
| Diastolic blood pressure (mmHg) | 89.0 (11.4) | 87.8 (10.0) | 81.3 (9.5) | 82.7 (9.6) |
| HbA1_c_ (%) | 6.7 (1.2) | 6.8 (1.5) | 6.7 (1.0) | 6.8 (0.9) |
| HbA1_c_ (%) | 50 (13) | 51 (16) | 50 (11) | 51 (10) |
| Total cholesterol (mmol/L) | 5.6 (1.0) | 5.7 (1.1) | 4.4 (1.0) | 4.4 (1.0) |
| Triglycerides (mmol/L) | 1.9 (1.2) | 2.0 (1.3) | 1.8 (1.2) | 1.8 (0.9) |
| HDL cholesterol (mmol/L) | 1.4 (0.4) | 1.4 (0.3) | 1.5 (0.5) | 1.4 (0.4) |
| LDL cholesterol (mmol/L) | 3.4 (1.0) | 3.5 (1.0) | 2.2 (0.8) | 2.2 (0.8) |
| Albumine Creatinine ratio (mg/g) | 3.8 (16.6) | 2.3 (7.4) | 6.2 (17.1) | 6.9 (28.5) |
| Any glucose-lowering drug | - | - | 100 (70.4) | 255 (75.4) |
| Metformin | - | - | 91 (64.1) | 222 (65.7) |
| Insulin | - | - | 18 (12.7) | 60 (17.8) |
| Sulphonylurea | - | - | 8 (5.6) | 26 (7.7) |
| Antihypertensives | 80 (45.7) | 141 (34.3) | 125 (88.0) | 285 (84.3) |
| ACE/ARB blockers | 39 (22.3) | 62 (15.1) | 111 (78.2) | 248 (73.4) |
| Betablockers | 38 (21.7) | 56 (13.6) | 50 (35.2) | 89 (26.3) |
| Calcium antagonists | 23 (13.1) | 38 (9.2) | 48 (33.8) | 126 (37.3) |
| Diuretics | 47 (26.9) | 76 (18.5) | 81 (57.0) | 171 (50.6) |
| Statins | 22 (12.6) | 56 (13.6) | 110 (77.5) | 268 (79.3) |
| Aspirin | 30 (17.1) | 39 (9.5) | 67 (47.2) | 185 (54.7) |
| Smoking |  |  |  |  |
| Non-smoker | 53 (30.3) | 157 (38.6) | 50 (32.5) | 144 (38.0) |
| Former smoker | 74 (42.3) | 144 (35.4) | 81 (52.6) | 183 (48.3) |
| Current smoker | 48 (27.4) | 106 (26.0) | 23 (14.9) | 52 (13.7) |

Categorical data are expressed as n (%), and continuous data as means (SD).
